# Supplementary material for: Broad-Spectrum Antiviral Activity of 3D8, a Nucleic Acid-Hydrolyzing Single-Chain Variable Fragment (scFv), Targeting SARS-CoV-2 and Multiple Coronaviruses In Vitro
Source: Viruses. 2021 Apr 9;13(4):650. doi: 10.3390/v13040650 (PMC8068894; doi:10.3390/v13040650)
Supplement: Supplementary file 1 [file viruses-13-00650-s001.zip › Supplementary Table 1.pdf]

Supplementary Data

Broad-spectrum antiviral activity of 3D8, a nucleic acid-hydrolyzing single chain variable fragment (scFv), targeting SARS-CoV-2 and multiple coronaviruses

Gunsup Lee[1¶], Shailesh Budhathoki[2¶], Geum-Young Lee[3], Kwang-ji Oh[1], Yeon Kyoung Ham[1], Young Jun Kim[1], Ye Rin Lim[1, 4], Phuong Thi Hoang[5], Yongjun Lee[5], Seok-Won Lim[6], Jun-Mo Kim[6], Seungchan Cho[3], Tai-Hyun Kim[1], Jin-Won Song[3], Sukchan Lee[5]* and Won-Keun Kim[2,4]*

[1]Novelgen Co., Ltd., R&D center, 77, Changnyong-daero 256 beon-gil, Yeongtong-gu, Suwon-si, Gyeonggi-do, Republic of Korea

[2] Department of Microbiology, College of Medicine, Hallym University, Chuncheon, Republic of Korea

[3] Department of Microbiology, Korea University College of Medicine, Seoul, Republic of Korea

[4] Institute of Medical Science, College of Medicine, Hallym University, Chuncheon, Republic of Korea

[5] College of Biotechnology and Bioengineering, Sungkyunkwan University, Suwon, Republic of Korea

[6] Animal Functional Genomics & Bioinformatics Lab., Department of Animal Science and Technology, Chung-Ang University, Anseong, Gyeonggi-do, Republic of Korea

Supplement table 1. Primers used for in vitro transcription viral spike gene amplification

Primer	Sequence	Type	Accession No.
**SARS-CoV-2**	5’- CAGATGCTGGCTTCATCAAA -3’	Forward	NC_045512
	5’- CATTGAGGCGGTCAATTTCT -3’	Reverse	
**HCoV-OC43**	5’- AGCTATACCCAATGGCAGGAA -3’	Forward	NC_006213
	5’- CCAGTGGTAATCGCTCCAC -3’	Reverse	
**PEDV**	5’- TGTCTCTGCGTCTTTTGGTG -3’	Forward	NC_003436
	5’- CTGCCACTTGCAGGATCATA -3’	Reverse	
