# Supplementary material for: Broad-Spectrum Antiviral Activity of 3D8, a Nucleic Acid-Hydrolyzing Single-Chain Variable Fragment (scFv), Targeting SARS-CoV-2 and Multiple Coronaviruses In Vitro
Source: Viruses. 2021 Apr 9;13(4):650. doi: 10.3390/v13040650 (PMC8068894; doi:10.3390/v13040650)
Supplement: Supplementary file 1 [file viruses-13-00650-s001.zip › Supplementary Table 2.pdf]

Supplement table 2. Primers used for RT-qPCR of SARS-CoV-2, HCoV-OC43, and PEDV

Primer	Sequence	Type	Location
**SARS-CoV-2 (N gene)**	5'-CACATTGGCACCCGCAATC-3'	Forward	28701-28719
	5'- GAGGAACGAGAAGAGGCTTG-3'	Reverse	28789-28808
**SARS-CoV-2 (E gene)**	5'-ACAGGTACGTTAATAGTTAATAGCGT-3'	Forward	26264-26289
	5'- ATATTGCAGCAGTACGCACACA-3'	Reverse	26306-26327
**SARS-CoV-2 (RdRp gene)**	5'- GTGARATGGTCATGTGTGGCGG-3'	Forward	15431 - 15452
	5'-CARATGTTAAASACACTATTAGCATA-3'	Reverse	15505 - 15530
**HCoV-OC43 (S gene)**	5'- AGTACTGCTATTTCTTGTTCAAACATGTG -3'	Forward	1123-1150
	5'- GCTCTACGACTTCGTCTGTTTTTAGA -3'	Reverse	1168-1193
**PEDV (N gene)**	5'- GATCGTGGCCGCAAACG -3'	Forward	26395-26411
	5'- TGCCATTGCCACGACTCC -3'	Reverse	26827-26844
